# Effectiveness of bone expansion, compacting and densification in narrow alveolar crests: a systematic review and a meta-analysis

**DOI:** 10.3389/fbioe.2025.1630495

**Published:** 2025-06-25

**Authors:** Nansi López-Valverde, Antonio López-Valverde, José Antonio Blanco

**Affiliations:** Department of Surgery, Faculty of Medicine, University of Salamanca, Salamanca, Spain

**Keywords:** narrow alveolar ridge, crestal expansion, threaded expanders, osteotomes, bone compaction, bone densification

## Abstract

**Background:**

The treatment of edentulism with dental implants is a common and reliable procedure with high medium- and long-term survival rates. Primary stability in the bone is vitally important to prevent micro-movements at the beginning of healing. However, the main drawback of dental implantology is bone deficiency. To alleviate this situation, clinicians’ resort to surgical techniques that increase bone volume and allow the devices to be placed. Bone expansion, compaction, and densification are used to compact the bone trabeculae, densifying the bone and improving the primary stability and osseointegration of implants. The aim of the study was to evaluate the role of these surgical techniques in deficient alveolar ridges in order to prepare them to receive durable dental implants.

**Methods:**

Searches were made of the PubMed/Medline, Embase, Cochrane Central, Dentistry and Oral Sciences Source and Web of Science (WOS) databases and GreyNet International, to identify RCTs, prospective studies, retrospective studies and case series published in English in the last 15 years, which evaluated the efficacy of bone expansion, bone compaction and densification in narrow alveolar ridges and their impact on bone density (BD), alveolar ridge expansion (CE) and implant stability quotient (ISQ). Methodological quality was evaluated using the Joanna Briggs Institute for RCTs (JBI MAStARI) tool and risk of bias using the Cochrane Risk of Bias Tool (RoB2), and meta-analyses were performed using Review Manager 5.4.1 software to calculate effect size and integrate the results of the included studies.

**Results:**

Ten of the 2,464 studies examined met the inclusion criteria. The meta-analysis of the parameters analyzed was favorable for the experimental group, indicating that bone expansion, compaction and densification techniques significantly increase DB (−0.71, 95% CI (Confidence Interval) [-1.15 to −0.27], p = 0.002), EC (−1.12, 95% CI [-2.21 to −0.03], p = 0.04) and ISQ (−8.88, 95% CI [-13.85 to −3.91], p = 0.0005), with a high publication bias for CE and ISQ.

**Conclusion:**

The techniques of bone expansion, compaction and densification demonstrated their effectiveness in narrow alveolar ridges, although studies are needed to validate the results found.

**Systematic Review Registration:**

Identifier CRD42025646738.

## 1 Introduction

The treatment of total or partial edentulism using dental implants has become common practice in dental surgeries and is currently considered a reliable and long-lasting surgical-prosthetic treatment, with survival rates estimated at over 90% over the first 10 years (Lekholm et al., 1999). The world market for dental implants is growing at a dizzying pace, with estimated figures in recent years exceeding 23 billion dollars (Alghamdi and Jansen, 2020).

**FIGURE 1:**
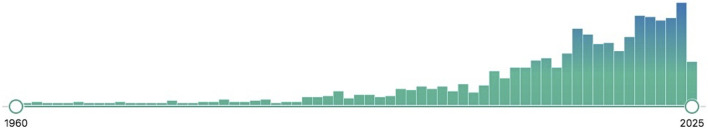
Graph of the increase in publications in recent years in PubMed using the term “ridge expansion”.

The need for implant treatments due to edentulism increases exponentially with age and, in this sense, it has been estimated that the aging of the population during the third decade of this century will reach figures of more than 20% of the total European population and 30% of the US population (Höpflinger, 2015). However, despite all the advantages they offer, according to longitudinal studies, a certain number of implants, which the scientific literature estimates at percentages of around 15%–19% for the maxilla and 1.2% for the anterior mandibular area, fail (el Askary et al., 1999a; el Askary et al., 1999b), despite the fact that Espósito et al. reported that biology-related implant failures, out of a sample of 2,812 implants, did not reach 8% in a 5-year follow-up period (Esposito et al., 1998). For the implant to be successful, there needs to be adequate bone compression around the device and immediate fixation at the moment of insertion (primary stability), as well as long-term fixation (secondary stability) (Misch et al., 2008). Primary stability is vitally important for long-term success, as it prevents micromovements of the implant during the early stages of the healing process. The degree of primary stability can be influenced by a series of factors, such as the design of the implant, the size of the osteotomy, bone density and/or the patient’s comorbidities (Heimes et al., 2023). It has been shown that high levels of primary stability at the time of implant insertion result in rapid secondary stability. Therefore, it is necessary to take maximum care of primary stability at the moment of insertion of the endosseous implant, to increase the chances of long-term permanence (Ivanova et al., 2021). It has been reported that the peri-implant bone must have a minimum thickness of 1.5 mm for the implant to support the loads (Chiapasco et al., 2009), however, bone deficit is the main drawback faced by dental implantology, and although 3D-printed scaffold-based technologies show promise for bone regeneration (Hao et al., 2024), failure rates in bone grafts are high due to insufficient blood supply necessary for integration and regeneration. When the alveolar crests lack adequate bone volume, additional surgical procedures are necessary to reconstruct and increase the bone deficit and, in this respect, several systematic reviews have identified the most appropriate techniques to provide the quality and quantity of alveolar bone necessary to allow the placement of a dental implant and, in addition, ensure its survival over time (Aghaloo and Moy, 2007; Aghaloo et al., 2016; Chiapasco et al., 2006). These reviews conclude that there is a certain discrepancy in the results of successes and failures of implants placed in surgically modified deficient ridges, with some indicating a higher failure rate (Crespi et al., 2021), others no significant differences (Elnayef et al., 2015) and others even reporting high success rates (Waechter et al., 2017; Anitua et al., 2013). This leads to a certain degree of confusion among clinicians, which means that making decisions based on scientific evidence when it comes to gaining bone volume in atrophic alveolar ridges is a complex task that is difficult to assess and predict. Furthermore, the variables change continuously, due, above all, to technological developments, the macro and micro design of the implants and the sophisticated diagnostic (Wang et al., 2025) and surgical techniques used. On the other hand, the avalanche and the large increase in publications in recent years on bone gain in atrophic edentulous ridges make it almost impossible to keep up to date with the latest techniques and appropriate instruments (Figure 1) and for all these reasons, the clinician must have the appropriate knowledge and criteria to put into practice the necessary surgical procedures that will provide the best results.

Horizontally deficient alveolar ridges are a common clinical situation, for which various procedures are performed to increase the crestal width: guided bone regeneration (GBR), division and expansion of the alveolar ridge, block bone grafts, etc. The division of the alveolar ridge was described by Tatum (1986), although it was Simion et al. (1992) who perfected it and published it in great detail in 1992. In 1994, Scipioni described the crestal expansion technique (CET) in edentulous jaws in a 5-year retrospective study on a large sample of 170 subjects and 329 implants (Scipioni et al., 1994). Subsequently, in 1999, he published a histological study on hard tissue repair in edentulous sites treated with the CET in 20 humans, suggesting that osteoblasts differentiate from pre-existing mesenchymal cells located in the original walls of the fissure, with the consequent deposition of new bone in the surgically created intraosseous defect (Scipioni et al., 1999).

“Bone compaction” (BC) and “bone densification” or “osteodensification” (ODT) are terms used to define a series of techniques that, through the use of certain surgical instruments, such as osteotomes, expanders, or specially designed surgical drills, generate compaction of the bone trabeculae. This produces tension from the osteotomy of the bone bed towards the outside, elastically deforming the bone through the tension produced by the osteodensifying surgical device, which, unlike perforating devices, does not cause bone loss. The elastic deformation of the bone would tend to return to its original shape when the tension disappears, increasing the original bone density. (Tricio et al., 1995).

All this, together with the incorporation of new techniques and equipment into clinical practice, would mean optimal use of alveolar ridges with bone deficiency, transforming them into useful sites for implant placement.

The aim of this systematic review and meta-analysis was to evaluate the effectiveness of bone expansion, compaction, and densification methods in narrow alveolar ridges to determine their usefulness in placing long-lasting dental implants, focusing on their impact on bone density (BD), alveolar crest expansion (ACE), and implant stability quotient (ISQ).

## 2 Methods

### 2.1 Presentation of the study

This systematic review has been carried out in accordance with the PRISMA (Preferred Reporting Items for Systematic Review and Meta-Analysis) criteria (Page et al., 2021) and the guidelines of the Clinical Practice Guidelines (Cumpston et al., 2019). The protocol for this systematic review has been registered in the PROSPERO database (International Prospective Register of Ongoing Systematic Reviews) with the registration number CRD42025646738.

### 2.2 Question of interest; PICOs format

The research question was approached according to the PICOs format: “Are methods of bone expansion, compaction or densification in narrow alveolar ridges, with horizontal atrophy, effective in promoting the long-term stability and durability of dental implants?”

To address the research question, intervention studies in adult patients with narrow maxillary and mandibular ridges were included (P), that evaluated or compared expansive treatment of bone compaction or densification (I), either with each other or with other surgical treatments (C), to observe the effects on the clinical parameters studied (O), considering only randomized clinical trials, case series, prospective studies and retrospective studies (s) (Table 1).

**TABLE 1:** PICOs format.

Population (P)	Patients with horizontal atrophy of the maxillary or mandibular ridges
Intervention (I)	Expansive treatments, bone compaction or densification
Comparison (C)	Between different techniques or expansive treatments for bone compaction or densification
Outcomes (O)	Clinical parameters: Δ Bone density (BD); Δ Alveolar ridge expansion (ARE); Δ ISQ (Implant Stability Quotient)
Type of studies (s)	Randomized clinical trials (RCTs), case series, prospective studies and retrospective studies

Δ, variable increase; BD, bone density; ARE, alveolar ridge expansion; ISQ, implant stability quotient.

### 2.3 Data sources and bibliographic search method

An electronic search was carried out in the PubMed/Medline, Embase, Cochrane Central, Dentistry and Oral Sciences Source databases and in the Web of Science (WOS) scientific information service to identify RCTs, prospective studies, retrospective studies and case series published in English in the last 15 years, using the EndNote bibliographic reference manager (Clarivate Analytics). We also searched the gray literature to obtain as much information as possible and avoid publication bias (GreyNet International). The Boolean operators AND and OR were used. The search strategy was designed using the terms described in Table 2.

**TABLE 2:** Search strategy.

Databases	Search terms
PubMed/Medline	Alveolar Process/surgery [MeSH term] OR Alveolar Ridge Augmentation/methods [MeSH term] OR Bone regeneration [MeSH term] AND Alveolar Ridge Augmentation [MeSH term] Jaw, Edentulous/surgery [MeSH term] OR Alveolar Bone Loss/pathology OR Alveolar Bone Loss/surgery OR Bone Resorption OR Bone Resorption AND Bone Remodeling/physiology AND Dental implants [MeSH term] AND Dental implantation AND Dental Implantation, Endosseous AND Mouth, Edentulous/surgery AND Osseointegration AND Osteotomes AND Osteodensification AND Bone densification AND Humans [MeSH term]
Embase	Narrow maxilla AND Alveolar Ridge Augmentation/methods [MeSH term] OR Bone regeneration [MeSH term]
Cochrane Central	Narrow maxilla AND Alveolar Ridge Augmentation/methods [MeSH term] OR Bone regeneration [MeSH term] OR Osteodensification OR Bone densification AND Humans [MeSH term]
Dentistry and Oral Sciences	Alveolar Ridge Augmentation AND Alveolar Ridge Augmentation OR Osteodensification OR Bone densification
Web of Science	Narrow maxilla AND Alveolar Ridge Augmentation/methods OR Osteodensification OR Bone densification AND Humans
Boolean operators	AND y OR

MeSH, Medical Subject Headings (MEDLINE, thesaurus).

### 2.4 Inclusion and exclusion criteria

The research studies were selected according to the following inclusion criteria: (1) randomized clinical trials (single or double blind), case series and prospective and retrospective studies that included more than 5 adult subjects (≥18 years of age) in the study; (2) with alveolar ridges with horizontal atrophy (dimension ≤2.5 mm); (3) that provided data on clinical parameters indicative of this anatomical limitation; (4) with statistical methods that included means and standard deviation, together with units with which to quantify bone surfaces or volumes; (5) published in English. Studies that did not follow all the criteria defined above were excluded, as were clinical cases, studies lacking data on crestal anatomical limitation, *in vitro* or animal experimental studies, literature reviews and irrelevant studies, such as editorials, conference contributions, etc.

### 2.5 Data extraction

Data from each included study were extracted and tabulated by two reviewers (NL-V and AL-V) using the standardized JBI-MAStARI data extraction tools. The titles and abstracts of the selected studies were reviewed by both reviewers. Those that met the inclusion criteria were read and the data extracted. The extracted data included specific details of the interventions, methods of delivery, populations, specific objectives, and significant results, in order to formulate the question of interest. Disagreements between reviewers were resolved through discussion and mediation by a third reviewer (JABR).

### 2.6 Evaluation of the quality of the results of the studies included

The studies included in this systematic review and meta-analysis were methodologically evaluated using the tool developed by the Joanna Briggs Institute for RCTs (JBI MAStARI), which adopts a particular point of view of the scientific evidence and the methods used to synthesize the different types of this evidence. The checklist consists of thirteen items and the responses to the items are either “yes”, “no”, “unclear” or “not applicable”. A “yes” response scores one point. To be considered a methodologically sound study, it must score at least seven points (Jordan et al., 2019).

### 2.7 Risk of bias

The Cochrane Risk of Bias Tool (RoB2) (Sterne et al., 2019), which assesses 7 domains of bias: random sequence generation (selection bias), allocation concealment (selection bias), blinding of participants and staff (performance bias), blinding of outcome assessment (detection bias), and incomplete outcome data (attrition bias), was used for their assessment. Studies were assessed with “high,” “low,” and “borderline” risk of bias; “borderline” risk of bias applied to those with a lack of information about possible bias.

### 2.8 Meta-analysis

The data were analyzed using Review Manager software (RevMan Software. Version 5.4.1, The Cochrane Collaboration, Copenhagen, Denmark; 2020). The efficacy of methods of bone expansion, compaction and densification in narrow alveolar ridges to assess their usefulness when installing dental implants in a lasting way was evaluated by means of a meta-analysis for each of the clinical parameters analyzed. Due to the heterogeneity of the results in the ISQ and CE variables, a meta-analysis of random effects and fixed effects was performed for the BD variable, given its homogeneity. All were based on the standardized mean difference (SMD) and the confidence interval (95% CI). Heterogeneity was considered low with I^2^ = 0–30%; moderate with I^2^ = 40–50%; substantial with I^2^ = 60–75%; and high with I^2^ ≥ 75%. The threshold for statistical significance was set at p < 0.05. No meta-analysis of adverse effects was performed due to the scarcity of reports on this topic.

## 3 Results

**FIGURE 2:**
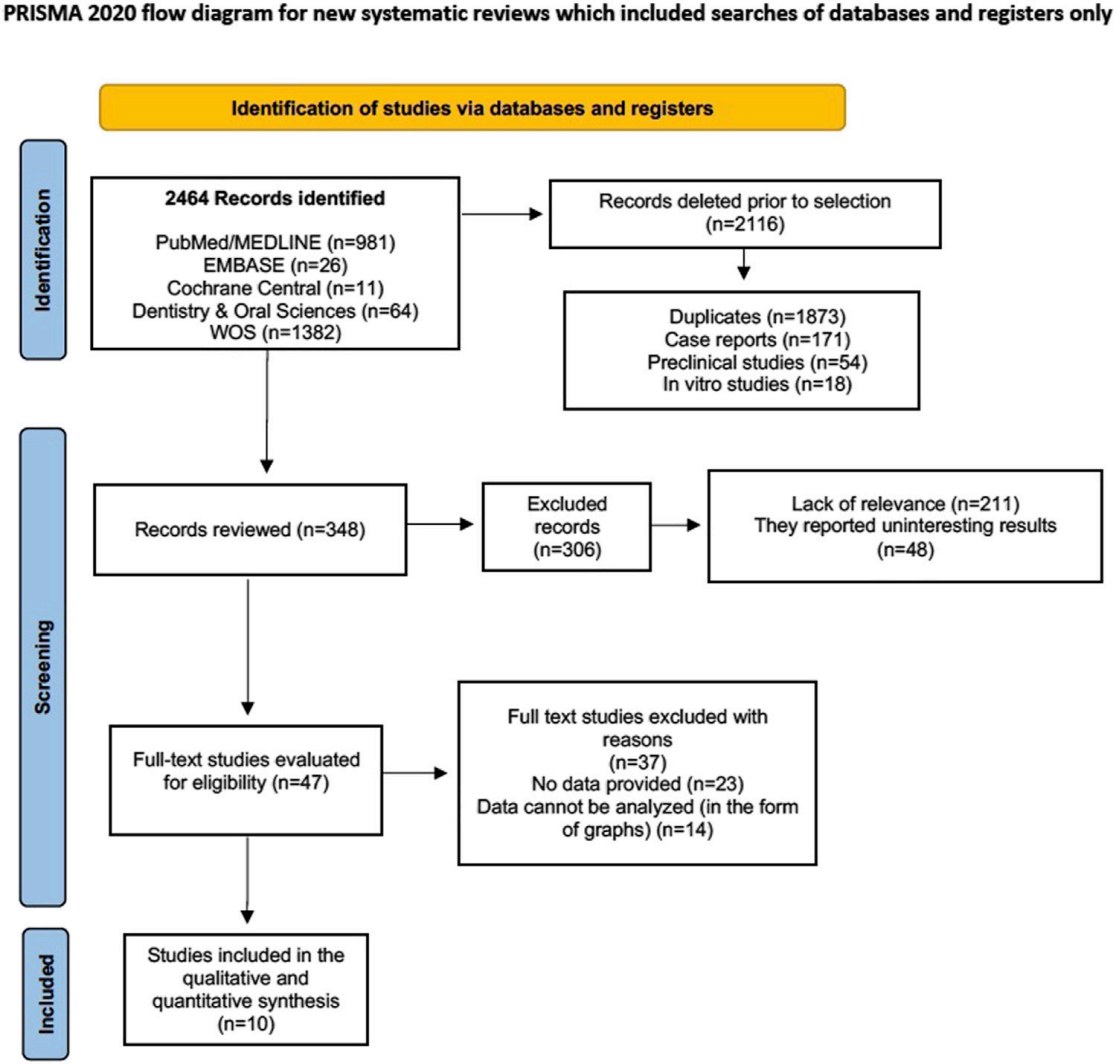
Flow chart.

A total of 2,464 records were originally identified (981 in PubMed/MEDLINE, 1,382 in WOS, 26 in EMBASE, 11 in Cochrane Central and 64 in Dentistry and Oral Sciences), and 2,116 duplicate records relating to clinical case reports, animal studies and *in vitro* studies were eliminated in an initial screening. In a second screening, a further 306 records were eliminated because they were considered irrelevant or because they reported data that was of no interest for the objectives set out in our study, leaving 47 studies to be evaluated and their eligibility determined. Of these, 37 were eliminated because they either did not provide data, or the data they did provide was in the form of graphs or figures that could not be analyzed mathematically/statistically, leaving 10 studies (Vaddamanu et al., 2024; Rizk et al., 2024; Tofan et al., 2024; Tushar et al., 2024; Ahmed et al., 2022; Salman and Bede, 2022; Yadav et al., 2022; Bergamo et al., 2021; Koutouzis et al., 2019; Anitua and Alkhraisat, 2016) to include in our systematic review and meta-analysis in synthesis, qualitative and quantitative (Figure 2).

### 3.1 General characteristics of the studies included

A total of 241 subjects were included in the 10 selected studies. Five studies were RCTs (Vaddamanu et al., 2024; Rizk et al., 2024; Tofan et al., 2024; Tushar et al., 2024; Ahmed et al., 2022), two were prospective studies (Salman and Bede, 2022; Yadav et al., 2022) and three were retrospective studies (Bergamo et al., 2021; Koutouzis et al., 2019; Anitua and Alkhraisat, 2016). Two studies assessed the bone density (BD in HU) (Vaddamanu et al., 2024; Ahmed et al., 2022), eight the CE (in mm) (Rizk et al., 2024; Tofan et al., 2024; Tushar et al., 2024; Ahmed et al., 2022; Salman and Bede, 2022; Yadav et al., 2022; Koutouzis et al., 2019; Anitua and Alkhraisat, 2016) and three recorded the ISQ values (Tushar et al., 2024; Ahmed et al., 2022; Bergamo et al., 2021). The follow-up periods ranged from the immediate assessment after the surgical technique to 6 months post-intervention. Only three studies were registered on the ClinicalTrials.gov RCT platform. The general characteristics of the included studies are shown in Table 3.

**TABLE 3:** General characteristics of the studies included.

Study and year	Type of study and registration	Number of participants	Clinical parameters analyzed	Follow-up period	Results
Vaddamanu et al. (2024)	RCT. ClinicalTrials.gov N°: NCT06268639	32	BD (in HU)	Assessment was immediate pre- and post-operatively	The observed increases in ODT, evidenced by the substantial changes in HU, demonstrate the effectiveness of ODT as a valuable technique in dental implantology. By promoting a more favorable bone environment for implant integration, ODT could lead to better outcomes for dental implants, especially in situations of low BD in HU (pre-operative 821.3 ± 485.5; post-operative 1,126.7 ± 373.6)
Rizk et al. (2024)	Comparative RCT. Not registered Ethics committee: Department of Oral and Maxillofacial Surgery of the Faculty of Dentistry at Ain Shams University	14	CE (in mm)	6 months	The change in bone width when comparing osteodensifying drills and implant expanders, immediately after the intervention, was greater with osteodensifying drills (p = 0.018 and p = 0.022, respectively). After 6 months, the difference between the two groups was also significant (p = 0.025 vs. p = 0.040)
Tofan et al. (2024)	Comparative RCT. Not registered. Approval of the institutional research ethics committee (reference number 410121). Implantology Unit/Department of Oral and Maxillofacial Surgery, Dental College Teaching Hospital, University of Baghdad, Iraq	19	CE (in mm)	Assessment was immediate pre- and post-operatively	There were no significant differences in the width of the alveolar ridge between the groups using osteodensifying drills and those using threaded expanders (5.48 ± 0.57 vs. 5.53 ± 0.71). However, the ODT technique using osteodensifying drills was much quicker to perform
Tushar et al. (2024)	Comparative study. Not registered. Institutional Ethics Committee of the Institute of Technology and Sciences. Dental College, Hospital and Research Center in Greater Noida, India. Approval number: ITSDCGN/2018/001	15	CE (in mm) Implant stability quotient (ISQ) BD (in HU)	6 months	The results for BD and ISQ were not significant. For bone measurements, the p-value was highly significant (p < 0.01)
Ahmed et al. (2022)	RCT. Not registered. Department of Oral and Maxillofacial Surgery of the Faculty of Dental Medicine of Al-Azhar University (Egypt)	20	Implant stability quotient (ISQ) and CE (in mm) BD (in HU)	6 months	The ODT zones showed a significantly higher insertion torque than the crestal split zones, however, the ODT showed a shorter duration than the crestal split. The horizontal bone gain in the postoperative period, at 6 months, did not show a significant difference (1.99 ± 0.68 vs. 2.63 ± 0.74)
Salman and Bede (2022)	Prospective study. Department of Oral and Maxillofacial Surgery, Faculty of Dentistry, University of Baghdad. Research Ethics Committee (protocol number: 207120). Registered at ClinicalTrials.gov (identification number: NCT04748952)	23	CE (in mm)	Assessment was immediate pre- and post-operatively	The average width of the bone before expansion was 4.04 ± 0.7 mm, while after expansion it was 5.3 ± 0.51 mm. The difference was statistically significant (p < 0.001)
Yadav et al. (2022)	Prospective study. No ethics committee appointment. Not registered	22	CE (in mm)	Assessment was immediate pre- and post-operatively	The preoperative bone width had an average value of 3.64 ± 0.41. After the operation, the bone width increased to an average value of 5.62 ± 0.45 mm. This postoperative increase was statistically significant (p < 0.001)
Bergamo et al. (2021)	Multicenter retrospective study. Ethics committees (protocol numbers #10295719.1.0000.5417 and #SH004 Integ Review). Clinical Trial Register (NCT04779203)	56	Implant stability quotient (ISQ)	6 months	The general ISQ data showed higher values for the experimental group (osteodensifying drills) compared to the control group (threaded osteotomes), regardless of the period evaluated (74 ± 1.5 vs. 66 ± 1.5). There were no significant differences in the mean expansion value in the apical zone between the groups; however, there was a difference in the crestal zone
Koutouzis et al. (2019)	Multicenter retrospective study. Not registered World Medical Association Declaration of Helsinki. All patients signed a consent form	21	CE (in mm)	Assessment was immediate pre- and post-operatively	The expansion did not show significant differences between the groups in the apical area (7.66 ± 1.41 vs. 8.66 ± 1.11). However, there was a significant difference in the crestal area. In group 1 (3–4 mm ridge) 75% expansion
Anitua and Alkhraisat (2016)	Retrospective study in accordance with the Declaration of Helsinki for research on human beings. Not registered	20	CE (in mm)	6 months	The preoperative width of the alveolar ridge was 3.1 mm (range, 2–6 mm) as measured on CBCT images. The surgical technique using osteotomes and transitional implants increased this width to 5.1 mm (range, 3.7–7.4 mm)

BD, bone density; HU, hounsfield unit; CE, crestal expansion; ISQ, implant stability quotient; ODT, osteodensification technique; CBCT, cone beam computed tomography.

### 3.2 Specific characteristics and sociodemographic data of the studies included

Two of the studies included were carried out in Egyptian centers (Rizk et al., 2024; Ahmed et al., 2022), two in Indian universities (Tushar et al., 2024; Yadav et al., 2022), two in Iraqi centers (Tofan et al., 2024; Salman and Bede, 2022) and the rest in Saudi Arabia, Brazil, United States and Spain [Vaddamanu et al., 2024; Bergamo et al., 2021; Koutouzis et al., 2019; Anitua and Alkhraisat, 2016, respectively]. The studies by Vaddamanu et al. (Vaddamanu et al., 2024) and Salman and Bede (2022) assessed the DO obtained using osteodensifying drills at the time of surgery and 6 months post-surgery. Five studies compared the DO obtained with osteodensifying drills and manual or motorized threaded expanders (Rizk et al., 2024; Tofan et al., 2024; Tushar et al., 2024; Ahmed et al., 2022). Bergamo et al. (2021) compared osteoconductive drills and conventional sequential drills in terms of DO. Yadav et al. (202)) assessed CE using manual threaded expanders and Anitua and Alkhraisat (2016) used motorized expanders to assess CE. Three studies evaluated the ISQ (Salman and Bede, 2022; Bergamo et al., 2021; Koutouzis et al., 2019) and most of the studies used CBCT as a radiological diagnostic tool (Rizk et al., 2024; Tofan et al., 2024; Tushar et al., 2024; Ahmed et al., 2022; Salman and Bede, 2022; Yadav et al., 2022; Koutouzis et al., 2019; Anitua and Alkhraisat, 2016). The specific characteristics of the studies are reflected in table 4.

**TABLE 4:** Specific characteristics of the studies included.

Study	Center and country	Anatomical characteristics of the subjects included	Surgical technique; equipment	Radiological analysis	Implant stability measurement
Vaddamanu et al. (2024)	King Khalid University. Saudi Arabia	People with partial or complete edentulous ridges in the maxilla or mandible	Osteodensifying drills (Huwais Technique)	Dentascan (CT) (DICOM software)	
Rizk et al. (2024)	Department of Oral and Maxillofacial Surgery at the Faculty of Dentistry, Ain Shams University. Egypt	Patients >18 years of age with totally or partially edentulous maxillary ridges, with horizontal bone defects, in which the bucco-palatal dimension >4 mm, with a minimum of 2 mm of trabecular bone core between the cortical plates and a height >10 mm	Osteodensifying drills (Huwais Technique) Manual threaded expanders	CBCT scan. (DICOM software)	
Tofan et al. (2024)	The Implantology Unit/Department of Oral and Maxillofacial Surgery, Dental College Teaching Hospital, University of Baghdad. Irak	Trabecular bone core ≥2 mm and trabecular/cortical bone ratio ≥1/1	Osteodensifying drills (Huwais Technique) Motorized threaded expanders	CBCT scan	
Tushar et al. (2024)	Institute of Technology and Sciences Dental College, Hospital and Research Center in Greater Noida. India	Absence of maxillary anterior teeth in the first and second quadrants with a horizontal width of bone >3–4 mm	Osteodensifying drills (Huwais Technique) Motorized threaded expanders	CBCT scan	
Ahmed et al. (2022)	Faculty of Dental medicine, Al-Azhar University. Egypt	Patients ≥18 years of age of both sexes with a long-standing edentulous area in the mandible that has healed for at least 6 months after extraction and an alveolar crest with horizontal dimensions of 3–6 mm width in the buccolingual direction and vertical dimensions >10 mm in height	Piezoelectric surgery with horizontal crestal incision. Osteodensifying drills (Huwais technique). Manual expanders	CBCT scan	
Salman and Bede (2022)	Department of Oral and Maxillofacial Surgery, College of Dentistry, University of Baghdad. Irak	Patients aged 18 years or over, with an alveolar crest width of 3–5 mm, with a trabecular bone core of ≥2 mm and a trabecular/cortical bone ratio of ≥1/1, as well as sufficient vertical dimensions	Osteodensifying drills (Huwais technique)	CBCT scan	ISQ. (Osstell Beacon^®^)
Yadav et al. (2022)	University of Medical Sciences, Saifai, Etawah, Uttar Pradesh. India	Patients aged between 20 and 60 with sufficient residual alveolar ridge height and width >3 mm	Threaded expanders	CBCT scan	
Bergamo et al. (2021)	Department of Prosthodontics and Periodontology, University of Sao Paulo, School of Dentistry. Brazil	It does not provide anatomical details of bone crests	Osteodensifying drills (Huwais technique) Conventional sequential drilling		ISQ (Osstell Mentor^®^)
Koutouzis et al. (2019)	Department of Periodontology, College of Dental Medicine, Nova Southeastern University, Florida. United States	It does not provide anatomical details of bone crests	Osteodensifying drills (Huwais technique)	CBCT scan	ISQ (Ostell, Gothenburg, Sweden^®^)
Anitua and Alkhraisat (2016)	Department Head, Eduardo Anitua Foundation, Vitoria, Spain	Horizontal bone atrophy in the jaw	Ultrasonic sagittal osteotomy. Motorized expanders (BTI Biotechnology Institute, Vitoria, Spain)	CBCT scan	

CBCT, cone beam computed tomography; DICOM, digital imaging and communications in medicine; ISQ, implant stability quotient.

### 3.3 Methodological rigor (JBI MAStARI)

**FIGURE 3:**
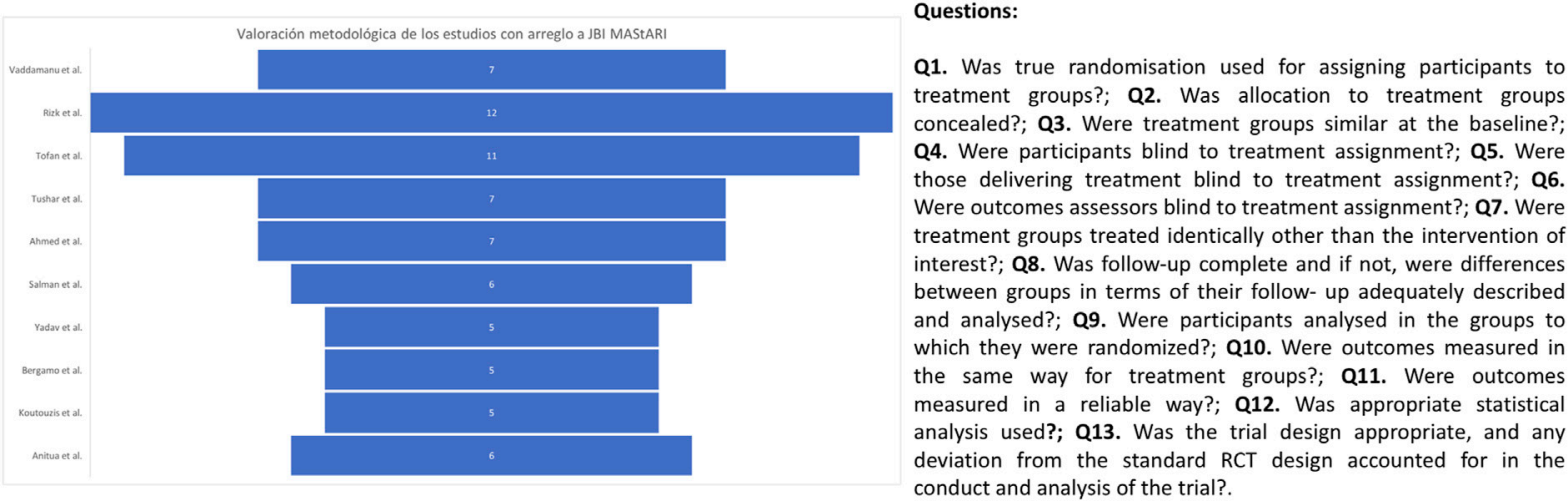
Methodological evaluation graph according to the Joanna Briggs Institute tool.

The methodological quality of the studies ranged from those that exceeded the 7 basic points considered to be of high/adequate methodological rigor (Vaddamanu et al., 2024; Rizk et al., 2024; Tofan et al., 2024; Tushar et al., 2024; Ahmed et al., 2022) to those that obtained a lower score (Salman and Bede, 2022; Yadav et al., 2022; Bergamo et al., 2021; Koutouzis et al., 2019; Anitua and Alkhraisat, 2016). However, the latter were prospective and retrospective studies and as such, lacked a control group; furthermore, they did not follow the criteria of randomization and blinding (Figure 3).

### 3.4 Overall meta-analysis

A meta-analysis of continuous variables was proposed that included means and standard deviations. Three individual meta-analyses were carried out according to the parameter analyzed: one for BD, a second for horizontal CE and a third for implant stability (ISQ), according to the surgical techniques proposed in the different studies. Heterogeneity ranged from 0% for the BD meta-analysis to 96% for the CE and ISQ meta-analyses, so a fixed-effect meta-analysis was performed for the BD variable and a random-effects meta-analysis for the ISQ and CE. No analysis of adverse effects was performed due to lack of data.

#### 3.4.1 BD

**FIGURE 4:**
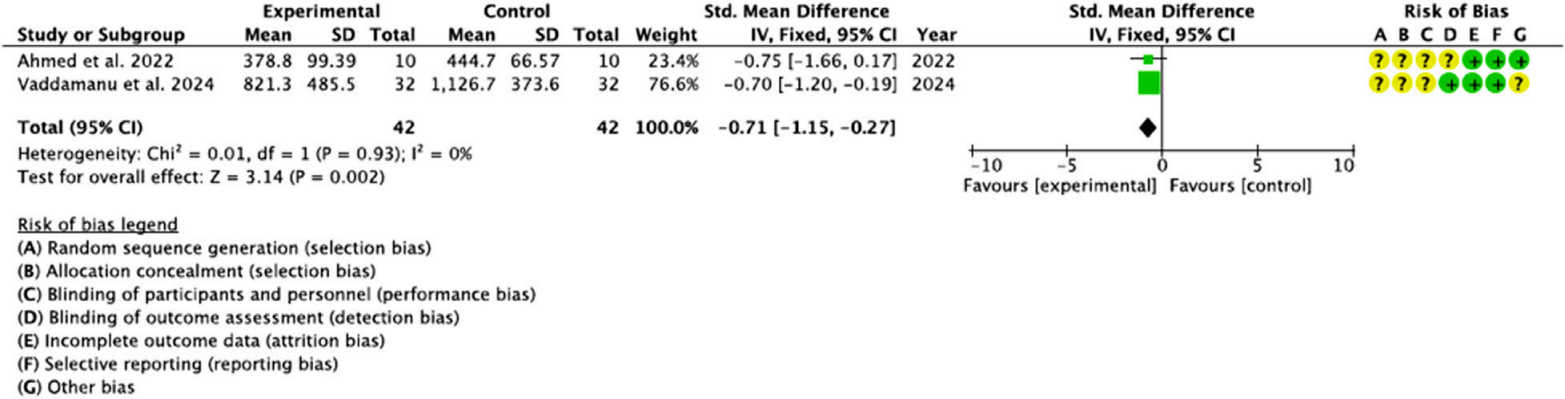
Forest plot for BD.

Two studies (Vaddamanu et al., 2024; Ahmed et al., 2022) provided data on the increases through osteodensification observed in BD. We identified no heterogeneity in the included studies (I^2^ = 0%). The meta-analysis showed a significant trend towards the experimental group compared to the control group (−0.71, 95% CI [-1.15 to −0.27], p = 0.002). (Figure 4).

#### 3.4.2 CE

**FIGURE 5:**
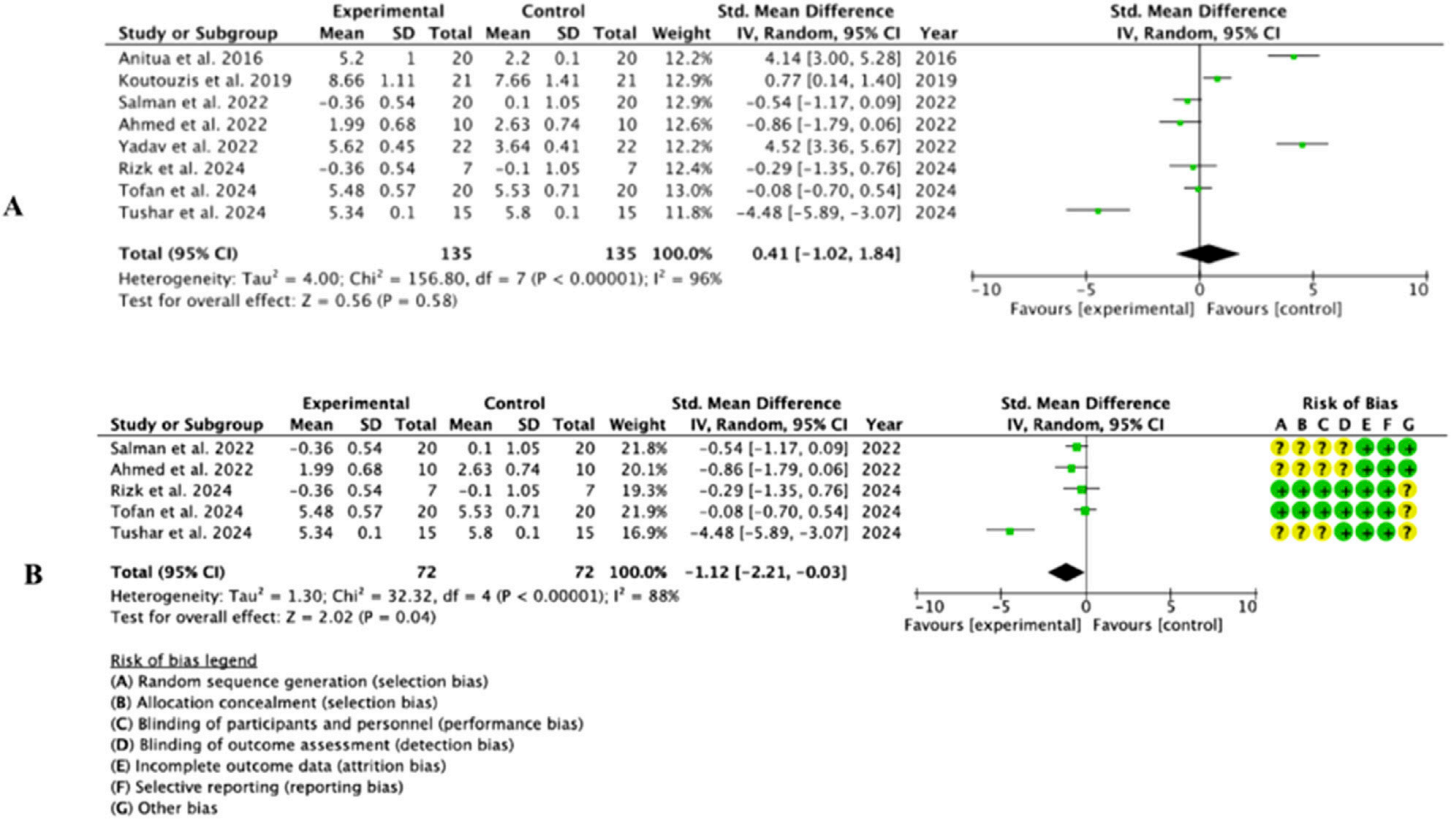
Forest Plot before **(A)** and after **(B)** the sensitivity analysis of the CE parameter.

Eight studies (Vaddamanu et al., 2024; Rizk et al., 2024; Tofan et al., 2024; Tushar et al., 2024; Ahmed et al., 2022; Salman and Bede, 2022; Koutouzis et al., 2019; Anitua and Alkhraisat, 2016) provided data on the gain in crestal width using different expansion techniques. Heterogeneity was high before and after the sensitivity test (I^2^ ≥ 75%). The meta-analysis before the sensitivity test was not significant (0.41, 95% CI [-1.02 to 0.84], p = 0.58); however, after the sensitivity test, once the studies by Yadav et al., Koutouzis et al. and Anitua and Alkhraisat (Yadav et al., 2022; Anitua and Alkhraisat, 2016) had been eliminated, it showed statistical significance in favor of the experimental group (−1.12, 95% CI [-2.21 to −0.03], p = 0.04). (Figure 5).

#### 3.4.3 ISQ

**FIGURE 6:**
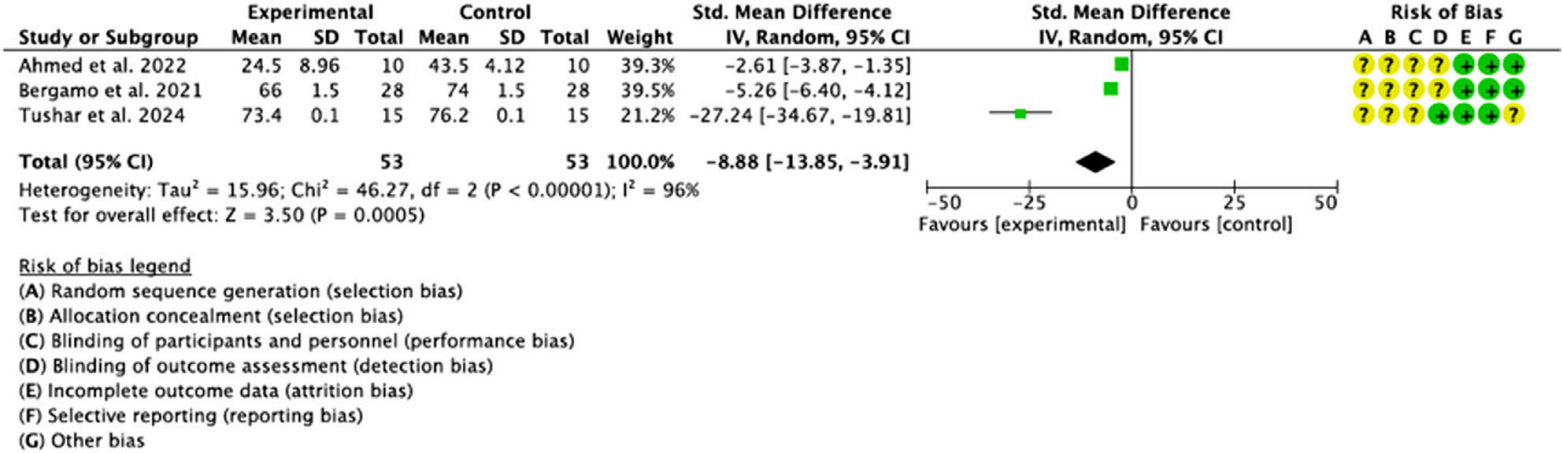
Forest Plot of the ISQ parameter.

Three of the studies included (Tushar et al., 2024; Ahmed et al., 2022; Bergamo et al., 2021) provided available data on the parameter of implant stability following expansion and ODT. Heterogeneity was high (I^2^ = 96%) and the meta-analysis showed a significant trend towards the experimental group compared to the control group (−8.88, 95% CI [-13.85 to −3.91], p = 0.0005). (Figure 6).

### 3.5 Risk of bias

**FIGURE 7:**
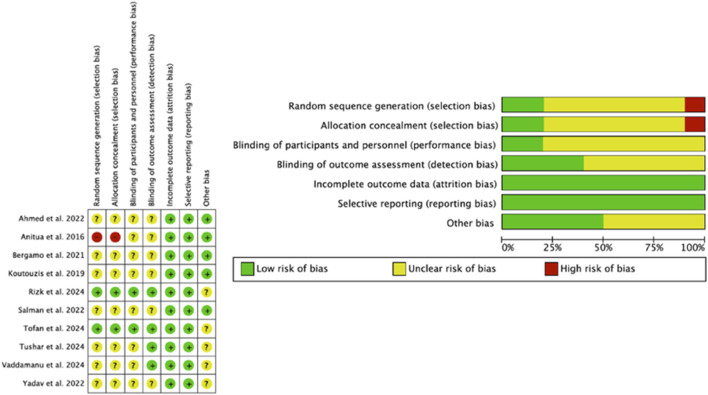
Graphic indicating risk of bias.

One of the pillars of evidence-based medicine is risk of bias and, therefore, the quality of the included studies was analyzed using the Cochrane Risk of Bias tool (Sterne et al., 2019). The studies were evaluated in 5 areas: (1) the randomization process; (2) deviations from planned interventions; (3) scarce or non-existent results data; (4) measurement of these results; (5) selection of the reported result and a sixth bias (6) which is related to the other biases. According to the Cochrane Handbook for Systematic Reviews of Interventions, a rating of “high” was given to studies considered to have a high risk of bias, “low” to those with a low risk of bias, and “borderline” to those with an unclear risk of bias or lack of information on possible bias. However, some studies included randomization software, and it was difficult to assess which domains they referred to and which they did not. The domains “random sequence generation” (selection bias), “allocation concealment” (selection bias), “blinding of participants and personnel” (performance bias) and “blinding of outcome assessment” (detection bias) were the ones with the greatest uncertainty. The study by Anitua and Alkhraisat (2016) was the one that presented the greatest risk, especially in domains 1 and 2 (selection biases) “random sequence generation” and “allocation concealment”, despite the authors recognizing among the limitations that, being a study with a retrospective design, it lacked a control group, and there was no randomization or blinding. The studies with the lowest risk of bias were those of Rizk et al. (2024) and Tofan et al. (2024) (Figure 7).

### 3.6 Publication bias

**FIGURE 8:**
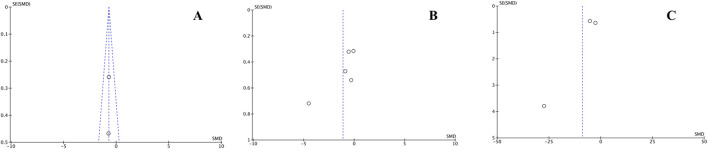
Publication biases expressed in funnel plots for the variables DO **(A)**, CE **(B)** and ISQ **(C)**.

The x-axis represents the observed results, and the y-axis represents the standard error. No dispersion was found in the funnel plot for BD (A), however, the plots for the variables CE and ISQ (B and C) showed a marked dispersion, which is evidence of a significant publication bias (Figure 8).

## 4 Discussion

In addition to systematically reviewing the current scientific literature, our study used a meta-analysis to evaluate the effectiveness of methods of bone expansion, compaction and densification in deficient alveolar ridges, in order to predict their usefulness when installing long-lasting dental implants. To increase the power of this systematic review, several types of studies were combined: RCTs, case series and prospective and retrospective studies. However, this particularity skews and limits the results. In fact, the studies with the worst evaluation were the prospective and retrospective ones that were not guided by randomization criteria; however, when the sample sizes of the primary studies are small, the combination of studies increases the statistical power.

The main result of our meta-analysis was that bone expansion, compaction, and densification methods in narrow alveolar ridges increase BD, ACE, and ISQ, making them useful for receiving dental implants.

A meta-analysis consists of conducting systematic reviews of quantitative and mathematical-statistical data from individual studies, using the data provided by the included studies, and calculating a statistical mean and an effect size for the event or treatment studied. Meta-analysis evaluates the need for future research (Sterne et al., 2019; Bown and Sutton, 2010), depending on the quality of the individual studies included (Straus and McAlister, 2000; Scheidt et al., 2019). Muka et al. (2020) proposed guidelines on how to conduct a systematic review and meta-analysis of observational studies and pooled RCTs, which served as a guide for our study.

Five RCTs (Vaddamanu et al., 2024; Rizk et al., 2024; Tofan et al., 2024; Tushar et al., 2024; Ahmed et al., 2022) and five observational studies (Salman and Bede, 2022; Yadav et al., 2022; Bergamo et al., 2021; Koutouzis et al., 2019; Anitua and Alkhraisat, 2016) met the inclusion criteria. The study by Tushar et al. (2024) found no differences in the ISQ between the groups comparing the motorized expanders and the osteodensifying drills; however, the motorized ridge expanders proved to be more effective in expanding the ridge, with a highly significant value (p < 0.01) and achieved, after 6 months, better primary stability than the ODT. These results would be contrary to those reported in the literature (Gaikwad et al., 2022; Lima Monteiro et al., 2024; Inchingolo et al., 2021).

The BD was evaluated by two RCTs (Vaddamanu et al., 2024; Ahmed et al., 2022) highlighting the benefits of ODT on BD and the primary stability of the implant, with the distal region of the osteotomy presenting the greatest BD (Vaddamanu et al., 2024). Ahmed et al. (2022) attribute the advantages of ODT to the autograft bone particles that would function as a nucleus for faster bone development around the implant, which could reduce healing time. However, Inchingolo et al. (2021) in a recent meta-analysis highlighted what we have emphasized about the existing contradictions in this regard in the literature. Some studies provide solid results that confirm the benefits of ODT, backed up by some statistically relevant values (Padhye et al., 2020; Hindi and Bede, 2020; Pai et al., 2018) and others, on the contrary, do not provide data that demonstrate differences in relation to the conventional technique. Koutouzis et al. in a retrospective multicenter study (Koutouzis et al., 2019) found no significant differences in the mean expansion value between the groups treated with and without ODT. Two preclinical studies in experimental sheep models (Lahens et al., 2019; Trisi et al., 2016) also found no differences in the osseointegration of implants placed with or without ODT. In this respect, the conclusions of a preclinical study published in the Journal of Dental Research (Wa et al., 2017) in a murine model are striking, providing evidence that condensation can increase the density of the peri-implant bone, although it does not guarantee greater bone-implant contact, nor does it improve implant stability. Compacted bone is denser, in the sense that it has a higher bone volume/total volume (BV/TV), however, this compacted bone would be structurally damaged and weakened (Zhang et al., 2021).

Tushar et al. (2024), Ahmed et al. (2022) compared variations in implant stability (ISQ index) in expanded bones in two RCTs; the first study using motorized expanders and the ODT, and the second using manual expanders and the ODT. Tushar et al. (2024) support the use of motorized expanders over bone densification, since, according to the authors, it limits CE. These conclusions would contradict certain reviews of the scientific literature that give greater popularity to the use of osteotomes, especially in the maxillary bone, due to the lower possibility of generating heat and the greater initial stability of the implant, due to lateral bone condensation (González-García et al., 2011; Jha et al., 2017). For their part, Ahmed et al. (2022) resorted to crestal corticotomy, combined either with manual expanders or osteodensifying drills, granting the latter technique greater implant stability, with results coinciding with those of Huwais and Meyer (2017). Bergamo et al. (2021) carried out a prospective multicenter study on 56 patients and 150 implants in several institutions, with a follow-up period of 6 months, and found that the ISQ values were higher for the experimental group (osteodensifying drills) compared to the control group (threaded osteotomes). However, despite the fact that implant insertion torque is the most common criterion for assessing adequate primary stability (Gallucci et al., 2018) and that low insertion torque (≤35 N force) is a controversial factor in the survival rate of dental implants, a recent systematic review with meta-analysis by Darriba et al. (2023) who evaluated the success rates at 24 months in 326 implants with immediate loading, concluded that a low insertion torque does not have a significant effect on implant survival rates. In this respect, another systematic review and meta-analysis carried out by Be et al. (2016) which included four studies in humans and six in animals, highlighted the lack of evidence in favor of high or low torque implants for our outcomes of interest: bone resorption, implant failure and BIC.

All these conclusions from the different studies lead us to consider the discrepancies that exist in the scientific literature on insertion torque values, from those who recommend low or moderate values, which allow the formation of considerable amounts of new bone that recovers around the implant (BIC, bone to implant contact) during the first stage of osseointegration (Duyck et al., 2015), to those who propose high insertion torques (>80 N) on the grounds that these high ISQ values would not cause bone resorption or implant failure (Consolo et al., 2013; Trisi et al., 2011). An example of this is the study by Khayat et al. (2013) who reported, in a prospective controlled clinical trial in humans, that the use of a high insertion torque of up to 176 N did not prevent osseointegration of the implant and did not cause marginal bone loss. Another recent comparative study (Arpudaswamy et al., 2024) that evaluated insertion torque and ISQ at the time of implant placement, and secondary stability metrics such as ISQ 3 months after insertion, between implants inserted into osteotomy sites prepared with conventional drills and osteosynthesis drills in the femoral condyles of New Zealand white rabbits, with low-density bone (type D4), showed that implants placed using ODT exhibited superior initial stability and superior stability progression, compared to those placed using conventional drilling techniques. Clinically, this would mean that ODT shows a higher insertion torque and a higher ISQ, by improving the bone density and volume surrounding the implants. This increased stability can lead to better osseointegration and a reduction in healing times, which ultimately benefits patients with compromised bone quality. On the other hand, Schierano et al., in a preclinical study published at the beginning of 2025 (Schierano et al., 2025), highlight the advantages of piezoelectric surgery in biological and clinical responses, especially in the increase of certain osteogenic factors and the formation of new bone, as well as a possible association with an increase in the ISQ.

CE was a clinical parameter evaluated in eight studies (Rizk et al., 2024; Tofan et al., 2024; Tushar et al., 2024; Ahmed et al., 2022; Salman and Bede, 2022; Yadav et al., 2022; Koutouzis et al., 2019; Anitua and Alkhraisat, 2016), resulting in the most highly valued of the three analyzed. Four of them were RCTs (Rizk et al., 2024; Tofan et al., 2024; Tushar et al., 2024; Ahmed et al., 2022) and four more (Salman and Bede, 2022; Yadav et al., 2022; Koutouzis et al., 2019; Anitua and Alkhraisat, 2016) were observational studies. Within the context of RCTs, Rizk et al. (2024), Tushar et al. (2024) and Ahmed et al. (2022) compared ODT with CE techniques, such as threaded expanders, either manual (Rizk et al., 2024), motorized (Tushar et al., 2024) or crestal corticotomy (“split”) (Ahmed et al., 2022). The results reported by these studies are completely contradictory. Rizk et al. (2024) analyzed fourteen implants placed in eight patients, seven using the osteodensifying drill technique and seven using expansion with manual threaded expanders. The analysis was carried out at three points: point 0 (below the implant cover screw), point A (1 mm below the level of the cover screw) and point B (2 mm below the level of the cover screw), in the immediate postoperative period and 6 months after placement. In the immediate postoperative period, they found a statistically significant difference between the two groups at points 0 and A in the experimental group (ODT) with the control group (manual expansion); however, the difference at point B was not statistically significant. Six months after insertion they found that the crest was undergoing continuous remodeling and, in some implants, the width of the alveolar crest was slightly less than the width of the preoperative alveolar crest. They even attribute the failure of three implants (despite the normal radiographic image of the surrounding bone) to the increase in bone density in the narrow alveolar crest, which could have caused a decrease in blood supply and an increase in temperature during surgery, for which reason they recommend using expansion techniques only in cases where other more predictable methods for horizontal bone augmentation are not feasible, such as GBR, bone blocks and crestal splitting. The conclusions of Rizk et al. (2024) are contradicted by those of Tushar et al. (2024), Ahmed et al. (2022) who propose expansion techniques (motorized expanders, ODT and split techniques) as effective methods for the expansion of narrow ridges. A systematic review carried out by Padhye et al. (2020) concluded by stating that, through ODT, bone expansion is achieved at the osteotomy site, although they recognize the need for well-designed prospective cohorts and randomized controlled trials to definitively establish the credibility of these techniques, both in the biological aspect and in clinical success. Similar conclusions are proposed by Inchingolo et al. (2021) who recommend thorough training of professionals before resorting to these techniques, considering them, from a practical point of view, complex to perform in inexperienced hands, describing the studies carried out to date as” modest and immature”, something we do not agree with, since the three studies discussed (Rizk et al., 2024; Tushar et al., 2024; Ahmed et al., 2022) achieved an adequate score on the JBI MAStARI scale of methodological quality, especially the study by Rizk et al. (2024) which achieved almost the maximum score.

The observational studies included (Salman and Bede, 2022; Yadav et al., 2022; Koutouzis et al., 2019; Anitua and Alkhraisat, 2016) evaluated 86 patients who received 119 implants with different conclusions that are presented and discussed below: Salman and Bede (2022) in a prospective study of 23 subjects, evaluated CE in narrow alveolar ridges, with low BD, before and after the use of osteodensifying drills, reaching the conclusion that the use of this type of device produced bone expansion without dehiscence or fenestration, increasing the width of the alveolar crest between 4 and 5.3 mm and allowing the simultaneous placement of implants with high primary and secondary stability. These results would be consistent with those obtained by Koutouzis et al. (2019) who, in a retrospective multicenter study of 21 subjects, also concluded that ODT allowed for an expansion of the alveolar crests in the coronal area of 3–8 mm (average 5.2 mm). Other studies (Tricio et al., 1995) also coincided in their results with the previous ones, reporting, through these expansion techniques, increases in crestal width ranging from 1.1 to 2.4 mm. Jarikian et al. (2021) in a non-randomized clinical study of 11 patients who had received 28 implants, compared ODT with bone densifying drills in narrow alveolar ridges (4–5 mm), obtaining similar results with an average of 2.36 mm of expansion, compared to the 1.5 mm achieved with threaded expanders. However, some preclinical studies have reported different results with regard to bone expansion, using ODT. Yeh et al. (2021) found that the ODT increased bone mineral density and primary contact between bone and implant in bovine rib segments; they also suggested that implant placement using ODT, compared to conventional drilling, can increase ridge dimensions in narrow alveolar ridges. Similarly, Li et al. (2023) demonstrated *ex vivo* that ODT increases the primary stability of cylindrical implants without overheating the bone and significantly increases the width of the crest. However, Tian et al. (2019), in atrophic mandibular ridges, in a porcine model, compared the ODT with the conventional osteotome technique, finding no significant differences between the two with regard to the degree of crestal CE. Yadav et al. (2022) assessed the stability of twenty-two implants placed in the maxilla and mandible of as many subjects after narrow ridge augmentation (assessed by CBCT), using the crestal split corticotomy technique. The preoperative bone width had an average value of 3.64 ± 0.41. After the operation, the bone width increased to an average value of 5.62 ± 0.45 mm, with a statistically significant postoperative period (p < 0.001). In this respect, a recent systematic review (Manekar et al., 2023) that analyzed twenty-two cohort studies and two randomized controlled trials, with 634 patients and 1,287 implants placed after alveolar ridge splitting and expansion, found an overall survival rate of 98.07% at 3 months of follow-up. They also showed that motorized expanders, as well as being a minimally invasive procedure, reduce the number of surgical procedures and the total duration of treatment and are recommended for osteocondensation. It should be noted, however, that the study by Yadav et al. (2022), together with Bergamo et al. (2021), Koutouzis et al. (2019) were the lowest rated in terms of methodology (5 points), as they did not report or left unclear issues of great relevance such as the randomization and blinding of participants and personnel involved in the study, which greatly limits the analysis of their results.

In a retrospective cohort study involving 20 patients and 31 implants (26 of which were placed in the posterior mandibular area), Anitua and Alkhraisat (2016) proposed the placement of transitional implants with a diameter of 2.5 and 3 mm, following CE with motorized threaded expanders, in crests with horizontal bone atrophy. In the second surgery, the transitional implant was removed, and the definitive dental implant was placed. Using CBCT, they verified that the preoperative width of the alveolar crest was originally 3.1 mm and the “guided bone augmentation” increased this width to 5.1 mm. This study reinforces the indication of crestal split in the treatment of horizontally atrophic jaws. Recently, a meta-analysis evaluated alveolar ridge splitting in implant surgery. This study by Lin et al. (2023) included twenty-four observational studies and one RCT; fourteen of the included studies investigated horizontal bone gain and seventeen examined implant survival. This meta-analysis found that the width obtained by crestal division was 3.633 mm within the range of 2.0–5.3 mm, which is consistent with what was reported by Anitua and Alkhraisat (2016). In addition, they found that the implant survival rate exceeded 98%, similar to the rate obtained with standard implant placement procedures. However, Crespi et al. (2021) in a retrospective study of thirty-eight patients, reported significantly lower incremental crestal values in the mandible than in the maxilla; nevertheless, the survival rates of the implants placed were 100% for the mandibles and 95.5% for the maxillas. The different expansion achieved in the mandible and maxilla could be explained by the fact that the buccal bone in the maxilla is highly viscoelastic and flexible, and this minimizes bone trauma. In contrast, the buccal wall of the mandible is made of harder, corticalized bone, which makes crestal division difficult. On the other hand, Altiparmak et al. (2017) had already reported that crestal splitting had a higher survival rate than onlay bone grafting (100% and 92%, respectively) and Mahmoud et al. (2020) had previously reported that there were no significant differences in the increase in crestal width between autologous bone block grafting and ridge splitting with flapless piezoelectric surgery. All of this would mean that the ridge splitting technique could shorten the treatment period, reduce postoperative inflammation and pain, eliminate the need for second surgeries, shorten treatment times and reduce costs. These considerations are at odds with the technique proposed by Anitua and Alkhraisat (2016) which, in addition to being complex and unsuitable for professionals without a certain level of experience, since the removal of the transitional devices can lead to bone fractures, lengthens the treatments and raises their cost, not exactly facilitating patient collaboration. Studies on new drilling techniques will help to alleviate many of these drawbacks (Fan et al., 2023).

Our study revealed a number of limitations that are worth highlighting: In terms of methodology, the heterogeneity of the studies included (RCTs and observational studies), together with publication and selection biases. Regarding the clinical aspect, the different surgical techniques and instruments used, the different methods of measuring the parameters analyzed, the different anatomical locations for expansion, and the follow-up periods.

All of this makes it hard to do a proper evaluation and means that the conclusions we present should be taken with some caution.

## 5 Conclusion

i) The two studies that evaluated BD were homogeneous, with a significant trend toward the intervention group.
ii) The three studies that evaluated ISQ, despite their heterogeneity, showed statistical significance toward the experimental group.
iii) The eight studies that evaluated CE, after sensitivity analysis, showed moderate statistical significance for the experimental group.

Despite the limitations, all this would mean that expansion, compaction and ODT in narrow alveolar ridges could be useful and reliable for clinicians when placing long-lasting dental implants, but more well-designed studies are needed to corroborate these results.

## Data Availability

The original contributions presented in the study are included in the article/supplementary material, further inquiries can be directed to the corresponding author.
